# Mobile Phones and Mental Well-Being: Initial Evidence Suggesting the Importance of Staying Connected to Family in Rural, Remote Communities in Uganda

**DOI:** 10.1371/journal.pone.0169819

**Published:** 2017-01-17

**Authors:** Amber L. Pearson, Elizabeth Mack, Judith Namanya

**Affiliations:** 1 Department of Geography, Environment & Spatial Sciences, Michigan State University, East Lansing, Michigan, United States of America; and Department of Public Health, University of Otago, Wellington, New Zealand; 2 Department of Geography, Environment & Spatial Sciences, Michigan State University, East Lansing, Michigan, United States of America; 3 Department of Geography, Environment & Spatial Sciences, Michigan State University, East Lansing, Michigan, United States of America; Peking University, CHINA

## Abstract

Due to the ubiquity of mobile phones around the globe, studies are beginning to analyze their influence on health. Prior work from developed countries highlights negative mental health outcomes related to overuse of mobile phones. However, there is little work on mental health impacts of mobile phone use or ownership in developing countries. This is an important gap to address because there are likely variations in mental health impacts of mobile phones between developing and developed countries, due to cultural nuances to phone use and distinct variations in financial models for obtaining mobile phone access in developing countries. To address this gap, this study analyzes survey data from 92 households in sparse, rural villages in Uganda to test two hypotheses about mobile phone ownership and mental health in a developing country context: (i) Mobile phone ownership is higher among more privileged groups, compared to less privileged groups (ie, wealth and ethnicity); and (ii) mobile phone ownership is positively associated with a culturally-relevant indicator of mental health, ‘feelings of peace’. Results indicate that households with mobile phones had higher levels of wealth on average, yet no significant differences were detected by ethnicity. As hypothesized, mobile phone ownership was associated with increased mental well-being for persons *without* family nearby (in the District) (p = 0.038) after adjusting for wealth, ethnicity and amount of land for crops and land for grazing. Mobile phone ownership was not significantly associated with increased mental well-being for persons with family nearby. These findings are consistent with studies of mobile phone use in other sub-Saharan African countries which find that phones are important tools for social connection and are thus beneficial for maintaining family ties. One might infer then that this increased feeling of mental well-being for persons located farther from family stems from the ability to maintain family connections. These findings are quite different from work in developed countries where mobile phone use is a source of technology-related stress or technostress.

## Introduction

Mobile phones are becoming increasingly common in poor regions of the world, and research from sub-Saharan Africa indicates that mobile phones may be very important tools for social connection [1], banking [2, 3] for accessing help and health care, income generation [4], safety [5] and even resistance and activism [6]. Other research on the continent shows that mobile phones have also been used by the health care and research community, to improve health care compliance [7], disease surveillance [8] and data collection [9].

The tremendous surge in mobile phone ownership offers the potential for health and social advantages to those who might not otherwise have access, thereby reducing vulnerabilities.

Work on health and mobile phones in developed and developing countries, however, emphasizes very different aspects of health. Recent studies of mobile and smartphone use in developed countries are beginning to assess mental health impacts of these technologies and find a variety of negative mental health impacts including sleep disruptions, depression, and psychological stress [10]. While studies in developed countries emphasize mental health impacts of mobile phone use, studies in developing countries emphasize their potential positive impacts on physical health [11–14].

To this point, studies of mobile phone use in developing countries have yet to assess their mental health implications. This is critical to examine given the rise in mobile phone ownership in developing countries, cultural differences in phone use [15, 16], and stark economic differences between residents of developed and developing countries. Economic differences mean many Africans share phones [2, 17] and use them on a pay-per-use basis [2, 18]. This type of use compared to single owner models and unlimited use via fixed monthly plans in some parts of the world, may reduce the frequency of phone use for residents in developing countries compared to their developed country counterparts. These differences in the intensity of mobile phone use are likely to produce variations in mental health impacts associated with mobile phone use. The ways in which mobile phones are also culturally embedded will produce differences in mental health impacts. The extent to which these economic and cultural differences produce variations in mental health impacts in residents of developing countries as compared to those of developed countries remains an understudied aspect of both mental health and mobile phone research.

Given this research need and the increasing prevalence of mobile phones in sub-Saharan Africa, this paper aimed to investigate the impact of mobile phone ownership (serving as a proxy for mobile phone use) on the mental health of residents of sparse, rural villages in Uganda. To do this, two hypotheses were tested: (i) Mobile phone ownership is higher among more privileged groups, compared to less privileged groups (i.e. wealth and ethnicity); and (ii) Mobile phone ownership is positively associated with a culturally-relevant indicator of mental health, ‘feelings of peace’, controlling for wealth, ethnicity and amount of land used for crops or grazing.

### Health implications of mobile phone use

The advent of mobile phones, and more recently smartphones, has increased access to information and our connectivity to people around the globe. In developing countries, mobile phones have enabled many places to leapfrog wired telecommunications services and become part of the informational economy [2]. Mobile phones have enabled participation in money transfer systems [2] such as the well-known M-Pesa application in Kenya [3, 19] for people living in regions with limited physical access to formal financial institutions. Mobile phones also offer great promise in improving the physical health of people in developing countries via a diversity of mhealth initiatives, ranging from reskilling healthcare professionals [20], to health education [2], to medical imaging [21]. For example, SIMpill is a mobile phone application that reminds patients to take medication [22]. Also, in Ghana, the Mobile Technology for Community Health initiative uses mobile devices to improve maternal health outcomes [11]. Several of these studies highlight the value of mobile applications to improving health outcomes in rural communities with lower access to health care facilities [11,13].

Recent work on mobile phone use and health has begun to explore the impacts of these devices on mental health. A key aspect of these studies is that most of them take place in developed countries. These studies find evidence of negative mental health impacts related to poor sleep [23, 24], information overload [25], and compulsive phone use [10]. Overall, these findings are indicative of ‘technostress’, which is defined as an inability to adapt to changes in computer-related technologies [10, 26]. Studies have also found that mobile phones are associated with addictive behaviors [27, 28].

Interestingly, results of mobile phone use studies in developing countries differ from studies of use in developed countries in two respects. First, they emphasize mobile applications for physical health issues [11–14]. Second, developing country studies find positive impacts of phone use on social cohesion [15]. In Africa, research found that mobile phones may serve as a substitute for physical trips to visit family [15, 29]. This is not to say that mobile phone use is entirely benign, however. There is some evidence that spending more money on phones negatively impacts family income and the ability to purchase basic life necessities [30].

In terms of specific impacts of mobile phones on mental health, it is anticipated that there may be differences between developed and developing countries for two reasons. First, the use of technology is subject to cultural appropriation [15]. This means that culture shapes the uses and impacts of technology. An example of the cultural context of mobile phone use in sub-Saharan Africa is the importance of kinship ties in social interactions [2]. In Burkina Faso, mobile phones are considered to be a socially meaningful form of communication, which is derived from the oral tradition in the country’s social structure, where traditions and respect are shared in oral exchanges [15]. This suggests that cultural views on mobile ownership and use in developing countries are likely different from typical developed country views of mobile phones as efficiency-enhancing gadgets.

Another important difference may be the financial burden mobile phone ownership places on residents of developing countries. For this reason, many people in Africa share phones and pay for services on a per-use basis [2]. These facets of mobile phone use, which place limits on the amount of use, may mitigate some of the mental health issues associated with overuse in developed countries. The extent to which these cultural and financial differences impact the linkages between mobile phone use and mental well-being in sub-Saharan Africa, remains unevaluated however. Given this research gap, the goal of this study is to undertake one of the first investigations of this linkage in an African context based on survey data from Kiruhura District of Uganda.

## Methods

### Ethical review

This study was approved by the University of Washington (HSD # 07-5209-J01), Mbarara University of Science and Technology and by the Uganda National Council for Science and Technology. Oral consent was granted by participants due to cultural norms and high levels of illiteracy. Consent was recorded as an X on the consent form, as approved by the ethical review boards above.

### Study area

This study takes place in the Kiruhura District of Uganda which represents a particularly interesting location for this research question. Uganda represents an understudied country in sub-Saharan Africa, in regards to mobile phone use, compared to Kenya [8,19], Burkina Faso [15], and Rwanda [17]. Uganda also lags several countries in sub-Saharan Africa in mobile phone adoption with just 52 mobile phone subscriptions per 100 people [31]. According to 2014 data from the World Bank on mobile phone subscriptions, Uganda ranked 37^th^ out of 48 countries in sub-Saharan Africa. It also ranked 231^st^ out of 250 countries globally for mobile phone subscriptions.

### Sampling

Within Uganda, this study was conducted in three villages in Kiruhura District (~4104 km^2^) [32], which consists primarily of sparse, rural villages in semi-arid savannah grasslands and scattered Acacia woodlands. The district is covered with low, undulating hills and wide valleys, with an average elevation of 1800 meters [32]. The main highway runs through this district, connecting the south-western parts of Uganda to the capital city, Kampala, the eastern regions and the Kenyan border.

Animal grazing and agriculture are two primary livelihoods, both largely subsistence. The primary livelihoods notionally translate to the two primary ethnic groups in the area, *Bahima* pastoralists and *Bairu* cultivators. Historically, inequality between these groups existed in three important ways: social, economic, and political. Most historians agree that before colonization, *Bahima* and *Bairu* were not socially equal. Because the two groups held very different occupations and engaged in separate economic activities, they were more or less coexistent subsistence systems. *Bairu* valued land for cultivation and *Bahima* valued cattle. Economic inequality surfaced through colonization and subsequent commodification schemes that re-assigned value through monetization. Cattle ownership emerged as a more lucrative investment, with rights to cattle ownership almost entirely claimed by *Bahima* [33]. Last, the pervasiveness of political inequality, whereby *Bahima* dominated *Bairu*, is consensual throughout all historical accounts [34–36]. This inequality persists today, in part due to the legacy of historical privilege in resource allocation to *Bahima* [37]. Therefore, we hypothesize that phone ownership will be significantly higher for *Bahima* compared to *Bairu*.

The sampling for this study first involved random selection of the three sub-counties in Kiruhura District. Then, one village was randomly selected from each sub-county, using a random number generator (Microsoft Excel Basic 2003, Redmond, WA). The 2012 census reports the sub-county populations to be 4200 (Kanyayeru), 13300 (Nyakashashara), and 16800 (Kitura), all with low population densities (<50 people/km^2^). Last, households were selected by simple random sampling from village rosters, using proportional population sampling from the three villages and totaling 100 households. There were no refusals to participate. However, from enrollment to the time of the survey, eight households were lost to follow-up as they moved outside the study area. Therefore, a total of 92 households were included in analyses.

### Mobile phone ownership and demographic data

We conducted a survey among the sampled households, which included questions related to household assets, whereby respondents listed the three most important assets. Respondents were probed about whether they owned a phone, at this time. If a mobile phone was owned, a 1 was recorded for the household and if not, a 0.

The surveys were conducted within each home, in *Runyankole*, with either the official or acting head of household (May 2009). The survey also included information about the household’s primary livelihood, ethnicity, self-reported wealth ranking, acres of land (used for crops and/or grazing), sanitation facilities, education level of both parents, primary expenditures, frequency of requests for assistance (financial or otherwise) to family members and/or others in the past year, and whether family lives within the District or beyond. The survey question was “Where is most of your family?” (see Supporting Information file: S1 Demographic survey). The word for family in *Runyankole* is *ab'eka yaawe* and its meaning includes both the nuclear and extended family. If the respondent had some family members within and some outside the District, it was up to the respondent to decide where ‘most’ family members live. A self-reported wealth ranking was used as one indicator of socio-economic status. In order to understand the relationship between self-reported wealth and other indicators of wealth, we first ranked the assets reported by households and assigned weights based on: the cash price, the indication of ‘luxury’ versus utility, and the importance of the item for livelihoods. Assets for livelihoods (eg nets, hoe, and milking pots) are valued very highly, even though they may not require large amounts of cash to purchase. This ranking was deemed suitable because, in preliminary analyses, a Spearman’s rank correlation of self-reported wealth was significantly, positively correlated with other indicators of wealth—the score of assets owned (rho = 0.26, p = 0.013) and amount of land owned (rho = 0.27, p = 0.011).

### Mental well-being data

One of the survey questions was, “What level of peace do you feel you have?” Options for responses ranged from ‘very high’ to ‘very low’, yielding values on a Likert-type scale from 1 to 5. The concept of ‘feelings of peace’ was defined previously, in 27 qualitative interviews conducted in the months prior to the survey. Interviews had been conducted among community members (selected based on ethnicity, age, gender and length of time living in the community) in an effort to increase ranges in responses. These interviews involved broader questions about the physical, social and built conditions of the community which may promote health. ‘Feelings of peace’ was an unexpected but common theme in many of the interviews, and is comparable to positive mental health, or mental well-being—hereafter mental well-being. While numerous mental health indices (eg, K-10) have been developed to measure poor mental health (ie, depressive symptoms, stress, anxiety) around the world, this measure was selected because of our interest in measuring positive mental health (or mental well-being) and because it has local relevancy in the study communities. Our survey question about feelings of peace is similar to the positive dimensions of the composite mental health index called MCS-12, which is used, in part, for monitoring emotional and mental health. Most questions in the MCS-12 are designed to indicate depressive feelings. However, our survey question most closely relates to the MCS-12’s question on positive emotional state, “*How much of the time during the past four weeks have you felt calm and peaceful*?”

### Statistical methods

To test the first hypothesis that mobile phone ownership is more prevalent in privileged groups (as indicated by wealth and ethnicity) we first examined household characteristics of our sample, split by phone ownership status. A chi-square test was used to test for significant differences between counts of households with and without phones according to ethnicity; a t-test was used to test for statistical differences in mean wealth by ownership. A multiple linear regression model was used to test the second hypothesis, that mobile phone ownership is positively associated with mental well-being ranked 1 to 5, where 5 represents higher levels of well-being). In this model, mental well-being was the dependent variable and phone ownership status (yes/no) was the independent variable of interest. Several control variables were used: the presence of family within the District, ethnicity and wealth. In terms of wealth, three measures were used: self-reported wealth level, amount of land used for grazing and amount of land used for crops. We also included an interaction term for phone ownership*family within the District. Regression coefficients are reported with associated 95% confidence intervals and p-values. All analyses were conducted using Stata v14 (College Station, TX, USA).

## Results

Table 1 shows household characteristics by phone ownership status. Of the 92 households included in this study, about 35% owned phones. Those with phones reported significantly higher levels of wealth, on average, compared to those without (p = 0.002), as hypothesized. Interestingly, those with phones had significantly more requests for help from others in the past year compared to those without phones (4 versus 2.3, p <0.001). Those with phones also had higher levels of mental well-being on average, although this difference was not statistically significant. Between ethnic groups, most *Bahima* and *Bairu* did not own phones, whereas half of households belonging to ‘other’ ethnic group owned a phone. Here it is important to note that the group ‘other’ included migrants from the Kampala area, the western districts and Rwanda. Contrary to our hypothesis, there was no statistically significant difference in phone ownership status by ethnicity (chi-square = 1.939, p = 0.379).

**Table 1:** Descriptive statistics of households by phone ownership status.

Household characteristics		Did not own phone n = 60	Owned phone n = 32	Total n = 92	p-value^†^
Level of wealth*, mean (sd)		2.7 (0.8)	3.3 (0.8)	2.9 (0.9)	0.002
**Number of requests for help from others last year, mean (sd)**		2.3 (2.3)	4.0 (1.4)	2.9 (2.6)	<0.001
**Level of mental well-being**^‡^**, mean (sd)**		3.4 (0.7)	3.7 (0.6)	3.5 (0.7)	0.056
**Ethnicity, n (%)**	Bairu	37 (69.8)	16 (30.2)	53 (57.6)	0.379
	Bahima	16 (64.0)	9 (36.0)	25 (27.2)	
	Other	7 (50.0)	7 (50.0)	14 (15.2)	
**Primary livelihood, n (%)**	Cultivation	35 (74.5)	12 (25.5)	47 (51.1)	0.020
	Cattle	18 (64.3)	10 (35.7)	28 (30.4)	
	Fishing	3 (27.3)	8 (72.7)	11 (12.0)	
	Other	4 (66.7)	2 (33.3)	6 (6.5)	
**Location of family, n (%)**	Beyond District	29 (58.0)	21 (42.0)	50 (54.3)	0.113
	In District	31 (73.8)	11 (26.2)	42 (45.7)	
**Sanitation type, n (%)**	Personal pit latrine	47 (66.2)	24 (33.8)	71 (77.2)	0.802
	Open defecation	2 (50.0)	2 (50.0)	4 (4.3)	
	Shared pit latrine	11 (64.7)	6 (35.3)	17 (18.5)	
**Primary expenditure, n (%)**	Animals	5 (31.3)	11 (68.8)	16 (17.4)	0.011
	Health	30 (77.0)	9 (23.1)	39 (42.4)	
	House	12 (92.3)	1 (7.7)	13 (14.1)	
	Food	8 (61.5)	5 (38.5)	13 (14.1)	
	Other	5 (45.5)	6 (54.6)	11 (12.0)	

*Level of wealth from 1 (low) to 5 (high)

^‡^Level of mental well-being from 1 (low) to 5 (high)

^†^p-values for differences calculated using a chi-square test for categorical measures and using a t-test for continuous measures

A low percentage of farmers (26%), followed by cattle keepers (36%) owned phones, whereas most fishermen did (73%). Of the 50 households who reported not having family in the District, just over half (58%) did not own a phone. Of the 42 households who reported having family in the District, most (74%) did not own a phone. Most households (77%) reported owning a pit latrine although there were no significant differences between sanitation types by phone ownership status. The largest percentage of households reported that their primary expenditures were on health, animals, housing and food, respectively. Of those whose primary expenditure was animals, most (69%) did not have a phone. Similarly, of those whose primary expenditure was health and food, most (77% and 62%, respectively) did not have a phone.

In regression modeling (Table 2), having a phone was associated with increased mental well-being for those *without* family in the District (p = 0.038) after adjusting for wealth, ethnicity and amount of land for crops and land for grazing. For those without a phone, having family within the District was associated with increased mental wellbeing, although this was not statistically significant (β = 0.26, 95%CI: -0.06–0.69, p = 0.107). We also detected significant, independent associations between mental well-being and wealth (β = 0.30, 95%CI: 0.13–0.46, p <0.001), land for grazing (β = 0.001, 95%CI: <0.001–0.001, p = 0.004) and ethnicity. In other words, increased mental well-being was associated with higher wealth and slightly more land for grazing. In addition, both ‘other’ ethnicities and *Bahima* had lower levels of mental well-being, compared to *Bairu*.

**Table 2:** Results for multiple linear regression model predicting mental well-being.

Predictors of mental well-being		β	95% CI	p
Phone ownership		**0.31**	**-0.06 0.69**	**0.099**
Family lives in District		0.26	-0.06 0.57	0.17
Phone ownership*Family lives in District		**-0.60**	**-1.17–0.03**	**0.038**
Level of wealth^§^		**0.30**	**0.13 0.46**	**<0.001**
Ethnicity	Bairu		*ref*	
	Bahima	**0.46**	**0.02 0.87**	**0.041**
	Other	**0.43**	**-0.07 0.80**	**0.021**
Land for grazing‡		**0.001**	**<0.001 0.001**	**0.004**
Land for crops‡		-0.004	-0.02 0.01	0.597

^§^ Level of wealth from 1 (low) to 5 (high)

^‡^ In acres

Items in bold font significant at the p<0.10 level

## Discussion

Our results indicated that existing social inequalities are partially reflected in phone ownership, whereby those with phones had higher levels of wealth on average. However, we did not detect significant differences in mobile phone ownership by ethnicity. Likely, the null findings for ethnicity relate to 1) the fact that the ‘other’ ethnic group consists primarily of servants who, while poor, earn cash and tend to purchase a phone; and 2) the generally low levels of ownership among both *Bairu* and *Bahima* and high levels of subsistence activities rather than cash-based activities in these groups. Our findings have been supported by other studies in sub-Saharan Africa. For example, a study conducted in Rwanda found that the majority of people who owned phones were from wealthier households than those who did not [17]. Since those with phones had significantly higher wealth than those without phones and since wealth was consistently associated with mental well-being, there is likely residual confounding related to socioeconomic status. However, because we found strong evidence of effect measure modification (the coefficients were in opposite directions) for those with family nearby and those without family nearby, it does not appear that wealth can solely explain the relationship between phone ownership and mental well-being.

In fact, those without family nearby who own phones reported significantly higher mental well-being (being at peace) than those phone owners who do have family nearby. This suggests that households without family nearby might rely on the phone as a way of staying connected to distant family members and may use it to make requests for help, as we also found that those with phones made significantly more requests for assistance from others than those without phones. These findings are supported by other work, hypothesizing that the maintenance of distant family networks through phone contact is a very important purpose of mobile phones, particularly in poor, remote settings [38], where many families are ‘stretched’ across rural and urban in an effort to diversify economic activities. Results of a study on gendered geographies of cell phone usage and significance in rural Kenya revealed that women who had family far away from where they lived used cell phones as a cheap and fast means of staying connected with their family which ensured peace of mind [39]. Another study [40] also found that parents reported that owning a mobile phone helped them keep in touch with their children which gave them peace of mind. Similarly, mobile phones have been shown to improve access to social support [41–43] and reduce loneliness and stress [44].

This positive link between mobile phone ownership and mental well-being seems to reflect a cultural importance of the phone as a way to maintain contact with distant family. Although this, too, is a use of phones in developed countries, the view of mobile phones is often quite different; they are seen as gadgets for enhancing efficiency. Study participants may also not experience some of the negative aspects of mobile phone use uncovered in developed countries, such as sleep disorders and compulsive use, because of the financial costs of using mobile phones in Africa. This means use is likely less intensive than in developed countries, and therefore not a source of technostress [10]. As the cost of phones decreases and the ubiquity of devices increases, further research could investigate if these ownership and use models change, and if these changes impact mental well-being. Additional research could evaluate the linkages between phone use and mental well-being in other countries in sub-Saharan Africa. Future research could usefully examine actual usage of the phone within the household, the purposes of making calls, and how calls may result in direct benefits to users, as our findings suggest that having a phone might improve both mental and physical health through access to family and others for assistance. We found that large proportions of households with primary expenditures on health and food were without phones. This could be explicitly investigated in future studies. Public-private partnerships may serve as one avenue to lower costs and thus increase access to mobile phones in developing countries, to ensure that mental health benefits are not only obtained by those who can afford a mobile phone, but also those for whom phones are less affordable. Such efforts must, of course, heed findings from developed countries on the potential negative mental health consequences of overuse.

It is important to note that this study had some limitations. First, households from villages were selected using population proportional sampling. In order to have more statistical power to conduct analyses by ethnicity, it would be useful to sample equally among the ethnic groups. Second, this study did not assess actual phone use or other related aspects, including phone sharing, reliability of network coverage, ability to charge a battery, affordability of airtime or the functionality of the phone itself. Likewise, this study did not measure differences in access to the phone within the household, as done in other Ugandan research which found that women did not have equal phone access [45]. The specific purposes of usage were also not examined and might be particularly important in these rural, remote settings—especially purposes related to transportation or the incidence of medical emergencies. Lastly, all the data on mobile phone ownership were self-reported which might lead to biased data. To overcome several of these limitations, future studies could usefully include either self-reported or objectively measured phone usage data, together with number of calls and locations dialled, as these data become increasingly accessible [46].

## Conclusions

It is evident that phone ownership reflects some existing social inequalities which is important to note given the findings of this study that phone ownership may be particularly beneficial to the mental well-being of households who lack nearby family networks. These findings provide initial evidence of the importance of staying connected to family for rural, remote communities in Uganda, suggesting that public health efforts can support the increase of equitable and affordable access to mobile phones in sub-Saharan Africa to enhance the connectedness and mental well-being of people, while paying attention to the potential negative mental health consequences of overuse.

## Supporting Information

S1 Demographic survey (PDF)
