# Supplementary material for: Mobile Phones and Mental Well-Being: Initial Evidence Suggesting the Importance of Staying Connected to Family in Rural, Remote Communities in Uganda
Source: PLoS One. 2017 Jan 17;12(1):e0169819. doi: 10.1371/journal.pone.0169819 (PMC5240969; doi:10.1371/journal.pone.0169819)
Supplement: S1 Demographic survey — (PDF) [file pone.0169819.s001.pdf]

1) DATE: ___________/ __________/ 2009 2) Surveyor: 3) HHID:

D E M O G R A P H I C   S U R V E Y

**Name of person surveyed** *(Last, First):*	**Participant ID:** -
**Ethnic Group**: ☐ Bahima ☐ Bairu ☐ Buganda ☐ Other ____	
**Education Level of mother:** ☐ None ☐ Primary ☐ Some Secondary ☐ Finished secondary ☐ Post-secondary ☐ DK ☐ NA	
**Education Level of father:** ☐ None ☐ Primary ☐ Some Secondary ☐ Finished secondary ☐ Post-secondary ☐ DK ☐ NA	
**Primary source of livelihood:** ☐ none ☐ Cultivation ☐ Cattle ☐ Business ☐ Civil servant ☐ Servant ☐ Fishing ☐ Other_______________	

H O U S I N G	
**Type of house**	Roof:☐ Thatch ☐ Iron sheet Floor:☐ Mud ☐ Cement<br>Walls:☐ Mud ☐ Brick ☐ Other_______
**Do you own your house?**	☐ N ☐ Y ☐ DK ☐ NA
**Number of houses on compound?**	________________
**L A N D**	
**Do you access land (graze, farm, live)?**	☐ N ☐ Y ☐ DK ☐ NA
**If YES, how would you classify the ownership of that land?**	☐ Private with title ☐ Ranch ☐ Private without title ☐ Communal<br>☐ Leasehold ☐ Other _____________
**If YES, what was/is the size?**	_____________ ☐ acres ☐ fields
**If YES, size used for grazing?**<br>**crops?**<br>**unused?**	_____________ ☐ acres ☐ fields<br>_____________ ☐ acres ☐ fields<br>_____________ ☐ acres ☐ fields
**IF YES CROPS GROWN, do you grow? (Check all that apply)**	☐ Banana ☐ Millet ☐ Maize ☐ Beans ☐ Cassava ☐ Irish
**If YES, is the quality of plot compared to your neighbors?**	☐ Very high ☐ High ☐ Average ☐ Low ☐ Very low ☐ None at all ☐ DK
**If YES, how did you acquire that land? (Check up to 3 that apply)**	☐ Inherited ☐ Bought ☐ Government ☐ DK ☐ Am servant ☐ Other _______
**W A T E R**	
**What is your level of access to water for HOME USE compared to your neighbors?**	☐ Very high ☐ High ☐ Average ☐ Low ☐ Very low ☐ None at all ☐ DK
**If YES ACCESS, who owned/owns that water?**	☐ Personal ☐ Shared ☐ Boss ☐ LMNP ☐ Public ☐ Other ________________
**If YES, is it?**	☐ Seasonal ☐ Permanent ☐ DK
**If YES, how many minutes does it take to get there, queue and return by foot?**	☐ <10 ☐ 11-29 ☐ 30-45 ☐ 46+
**If YES, PRIMARY WET SEASON source for HOME WATER?**	Name: ____________________<br>☐ Borehole ☐ Farm pond/dam ☐ Rainwater ☐ Valley tank ☐NA<br>☐ Government dam ☐ Other ______________
**If YES, in the past 2 weeks has the water at this source been unavailable for 1 whole day?**	☐ N ☐ Y ☐ DK ☐ NA
**If YES, PRIMARY DRY SEASON source for HOME WATER?**	Name: ____________________<br>☐ Borehole ☐ Farm pond/dam ☐ Rainwater ☐ Valley tank ☐NA<br>☐ Government dam ☐ Other ______________
**If NONE AT ALL or IN TIMES OF DROUGHT, how do you COPE with HOME WATER SCARCITY? (Check up to 3 that apply)**	☐ Buy water ☐ Trade for water ☐ Migrate ☐ Lk Kakyera ☐ Neighbor sneak ☐ Neighbor permis ☐ Lk Mburo sneak ☐ Lk Mburo permis ☐ Other _____________ ☐ NA
**What is your level of access to water for LIVESTOCK compared to your neighbors?**	☐ Very high ☐ High ☐ Average ☐ Low ☐ Very low ☐ None at all ☐ DK ☐NA

1) DATE: ___________/ __________/ 2009 2) Surveyor: 3) HHID:

**If YES ACCESS, who own that water?**	☐ Personal ☐ Shared ☐ Boss ☐ Public ☐ National Park ☐ King ☐ Other _____________
**If YES, how many minutes does it take to get there, queue and return by foot?**	☐ <10 ☐ 11-29 ☐ 30-45 ☐ 46+
**If YES, PRIMARY WET SEASON source for LIVESTOCK WATER?**	Name: _____________________ ☐NA<br>☐ Farm pond/dam ☐ Rain ☐ Lk Kakyera ☐ Valley tank ☐ Govt dam ☐ Lk Mburo
**If YES, PRIMARY DRY SEASON source for LIVESTOCK WATER?**	Name: _____________________<br>☐ Borehole ☐ Farm pond/dam ☐NA ☐ Rainwater ☐ Valley tank ☐ Govt dam ☐ Lake Mburo ☐ Lake Kakyera
**If NONE AT ALL or IN TIMES OF DROUGHT, how do you COPE with LIVESTOCK WATER SCARCITY? (Check up to 3 that apply)**	☐ Buy water ☐ Trade for water ☐ Migrate ☐ Lk Kakyera ☐ Neighbor sneak ☐ Neighbor permis ☐ Lk Mburo sneak ☐ Lk Mburo permis ☐ Other _____________ ☐ NA
**F O O D**	
**What is your level of FOOD SCARCITY compared to your neighbors?**	☐ Very high ☐ High ☐ Average ☐ Low ☐ Very low ☐ None at all ☐ DK
**If YES SCARCITY, how do you COPE with FOOD SCARCITY? (Check up to 3 that apply)**	☐ Buy food ☐ Trade ☐ New crops ☐ Exotic cows ☐ Migrate ☐ Fence land ☐ Gov't asst. ☐ Help: family in comm. ☐ Help: family outside ☐ Help: neighbor ☐ Kill cow ☐ Remain ☐ Other _______________
**If YES, reasons for food insecurity? (Check up to 3 that apply)**	☐ Lack of land ☐ Drought ☐ Theft ☐ Wild animals ☐ Wandering ☐ Animal diseases ☐ Crop diseases Other _______________
**Number of typical meals/meals yesterday?**	☐ 0 ☐ 1 ☐ 2 ☐ 3+ ☐ DK
**E C O N O M I C S**	
**Primary sources of expenditure: (Check up to 3 that apply)**	☐ Health ☐ Servnts ☐ Clthes ☐ Tranprt ☐ Animals ☐ Taxes ☐ Leisure ☐ DK ☐ House items ☐ Alcohol ☐ Cosmetics ☐ Other ______________ ☐ Food
**Do you own livestock?**	☐ N ☐ Y ☐ DK ☐ NA
**Number of children currently in school?**	___________________
**How wealthy were/are you compared to your neighbors?**	☐ Very high ☐ High ☐ Average ☐ Low ☐ Very low ☐ None at all ☐ DK
**P O L I T I C A L / S O C I A L P A R T I C I P A T I O N**	
**Asked help from non-family last yr?**	☐ 0 ☐ 1 ☐ 2 ☐ 3+ ☐ DK
**Asked help from family last yr?**	☐ 0 ☐ 1 ☐ 2 ☐ 3+ ☐ DK
**Level of attendance of LC1 meetings by your hh?**	☐ Very high ☐ High ☐ Average ☐ Low ☐ Very low ☐ None at all ☐ DK
**Someone from hh in a position of leadership?**	☐ N ☐ Y ☐ DK ☐ NA
**Where is most of your family? (Check most precise geography)**	☐ District ☐ Out of Dist. ☐ Out of UG ☐ NA ☐ DK
**How is your level of peace?**	☐ Very high ☐ High ☐ Average ☐ Low ☐ Very low ☐ None at all ☐ DK

**Method of drinking water treatment:** ☐ Chlorine ☐ Boil ☐ Iodine ☐ Filter ☐ NA ☐ Other ______________________	**Cleaned water container in last week?** ☐ 1 ☐ 2 ☐ 3 ☐ 4 ☐ 5 ☐ 6 ☐ Daily ☐ None ☐ NA
**Amount of water collected between today and yesterday?** ____________ ☐ Small ☐ Large **JERRYCANS**	
**Type of fuel used for cooking**: ☐ Kerosene ☐ Electricity ☐ Charcoal ☐ Wood ☐ Dung ☐ NA ☐ Other____________________________	
**Location of cooking:** ☐ Room for sleeping ☐ Other room in house ☐ Detached building ☐ Outdoors ☐ NA	
**Type of toilet:** ☐ Pit latrine ☐ Open defecation ☐ Other_________________	**Location of toilet:** ☐ Within property ☐ Outside property ☐ NA

N o t e s :
